# Impact of Rehabilitation on Outcomes after TAVI: A Preliminary Study

**DOI:** 10.3390/jcm7100326

**Published:** 2018-10-05

**Authors:** Christian Butter, Jessica Groß, Anja Haase-Fielitz, Helen Sims, Cornelia Deutsch, Peter Bramlage, Michael Neuss

**Affiliations:** 1 Department of Cardiology, Heart Center Brandenburg, Bernau 16321, Germany; j.gross@immanuel.de (J.G.); a.haase-fielitz@immauel.de (A.H.-F.); m.neuss@immanuel.de (M.N.); 2 Brandenburg Medical School (MHB) “Theodor Fontane”, Neuruppin 16816, Germany; Christian.butter@mhb-fontane.de (C.B.); anja.haase-fielitz@mhb-fontane.de (A.H.-F.); 3 Institute for Pharmacology and Preventive Medicine, Cloppenburg 49661, Germany; helen.sims@ippmed.de, helenmsims@yahoo.co.uk (H.S.); cornelia.deutsch@ippmed.de (C.D.); peter.bramlage@ippmed.de (P.B.)

**Keywords:** cardiac rehabilitation, TAVI, mortality, aortic stenosis, heart valve

## Abstract

The benefit of rehabilitation in elderly patients undergoing transcatheter aortic valve implantation (TAVI) for treatment of severe aortic stenosis is unknown. The impact of declining rehabilitation programs on mortality has also not been described. In a longitudinal cohort study of 1056 patients undergoing elective TAVI between 2008 and 2016, logistic regression analysis was used to assess the relationship between treatment modality and outcome according to whether or not patients participated in a three-week rehabilitation program after TAVI. Subgroup analyses included patient outcome separated according to cardiac, geriatric, or no rehabilitation. A total of 1017 patients survived until hospital discharge (96.3%) and were offered rehabilitation, 366 patients (36.0%) declined to undergo rehabilitation, with the remaining patients undergoing either cardiac (*n =* 435; 42.8%) or geriatric rehabilitation (*n* = 216; 21.2%). Mortality at six months was lower for patients receiving rehabilitation compared with those who had not (adjusted odds ratio (OR): 0.49; 95% confidence interval (confidence interval [CI]: 0.25–0.94; *p* = 0.032). Sub-analysis showed the benefit of cardiac (adjusted OR: 0.31; 95% CI 0.14–0.71, *p* = 0.006), but not geriatric rehabilitation (adjusted OR 0.83; 95% CI 0.37–1.85, *p* = 0.65). A program of rehabilitation after TAVI has the potential to reduce mortality. Future studies should focus on health-orientated behavior and identifying risk factors for declining rehabilitation programs.

## 1. Introduction

Cardiac rehabilitation programs improve functional capacity and quality of life in patients with aortic valve replacement [1]. The development of transcatheter aortic valve implantation (TAVI) has provided an alternative to open heart surgery for patients with severe aortic stenosis who are at high risk for periprocedural mortality. Mortality after TAVI continues to decrease over the last few years and equals that of surgical aortic valve replacement in selected patients [2]. Patients who undergo TAVI are older and frailer than those that undergo surgical valve replacement and usually present with more comorbidities. This can result in a significant burden for follow-up care, despite the minimally invasive nature of the transcatheter procedure. In view of an aging global population and the trend toward performing TAVI in elderly patients at lower risk, this burden is set to increase. Therefore, strategies aimed at improving mid- and long-term outcomes after TAVI are essential.

To date, approaches to encourage post-TAVI recovery have included early mobilization, exercise training, patient education, and psychological support [3,4,5,6]. A number of studies demonstrated certain benefits of multicomponent rehabilitation programs for TAVI patients [1,4,7]. These have generally focused on functional and emotional status, with no evaluation of how the programs translate into long-term health outcomes. Furthermore, data comparing patients who have undergone rehabilitation with those who have not are lacking, implying that the contribution of aortic stenosis symptom alleviation cannot be distinguished. In Germany, rehabilitation after TAVI is widely available; however, a proportion of patients decline to undergo such follow-up care [4,8]. Here, we evaluate differences in outcomes, including six-month mortality for patients with and without rehabilitation after TAVI—with a special focus on patients after cardiac or geriatric rehabilitation.

We hypothesized a rehabilitation program would produce reduced cardiovascular mortality.

## 2. Material and Methods

### 2.1. Data Source and Study Population

All patients undergoing elective TAVI at the Brandenburg Heart Centre in Bernau, Germany between 2008 and 2016 were enrolled and prospectively followed by telephone calls or letters. Subjects were excluded if they died during initial hospital stay. Therefore, hospital discharge was considered as baseline for further analyses.

The study was performed in accordance with the Declaration of Helsinki. The Strengthening the Reporting of Observational Studies in Epidemiology guidelines for reporting observational studies was used [9].

The ethics committee advised that no formal vote and no written informed consent beyond the agreement at hospital admission was necessary because data collection and assessment was part of hospital-wide measure of quality management.

### 2.2. Data Recorded

Patient characteristics were recorded at baseline, including demographics and comorbidities. A full cardiac history was taken and the logistic EuroScore I was calculated. Echocardiographic parameters were documented, including left ventricular ejection fraction (LVEF), and New York Heart Association (NYHA) class was determined. Levels of N-terminal brain natriuretic peptide (NT-proBNP) were measured. Age was analyzed by one year increments, body mass index (BMI) by a 1 kg/m^2^ increase, glomerular filtration rate [GFR] was considered as less than vs. 60 or more mL/min, LVEF by an increase of 1%, the EuroScore by an increase of 1%, and the remaining variables as yes vs. no.

We chose 6-month mortality as the follow-up period to discriminate potential intermediate effects of a rehabilitation program from comorbidities becoming more relevant for long-term patient outcome, such as for 1-year mortality.

During the TAVI procedure, complications were documented. Prior to discharge, a full cardiac assessment was performed including aortic valve function, LVEF, and NT-proBNP levels.

### 2.3. Follow-up

Patients were subsequently evaluated at an outpatient appointment 6 months after TAVI. Also, the patients’ vital status was assessed by telephone or letter. If no reply was received, the family doctor was contacted by telephone. Mortality and rehospitalization since discharge were documented and cardiac function was again assessed.

### 2.4. Rehabilitation

In accordance with standard clinical practice in Germany, all patients were offered rehabilitation after the TAVI procedure in preparation for hospital discharge. Those considering post-interventional rehabilitation programs either underwent cardiac or geriatric rehabilitation on an inpatient basis for approximately 3 weeks. The rehabilitation centers (*n =* 4) were representative for post-interventional rehabilitation programs. The components of the rehabilitation included patient health education, advice on cardiovascular risk reduction, including lifestyle and dietary advice, psychological support, and physical activity.

As is the case with cardiac rehabilitation, geriatric rehabilitation is provided by a team of health care professionals including physicians, physiotherapists, occupational therapists, and respiratory therapists with multidimensional assessment tools.

### 2.5. Geriatric Rehabilitation

Geriatric rehabilitation is a multi-professional, interdisciplinary concept. Geriatric rehabilitation aims to increase the level of functional independence, social inclusion, and to return the patient to a pre-injury quality of life.

Characteristics of geriatric rehabilitation include a variety of patient-adapted therapeutic approaches, interdisciplinary teamwork, active and holistic care, and consideration of somatic, psychologic, and social aspects within a pre-set treatment plan. At baseline, the condition of the patient is assessed and monitored during the course using evaluation tools like the Barthel index, time-up-and-go-test, Tinetti-test, and more. During geriatric rehabilitation, self-help measures are promoted.

In comparison with patients within “indication-specific rehabilitation programs”—such as cardiac rehabilitation—geriatric patients are in need of help and have lower physical, mental, cognitive, and psychologic capacities. Even during the process of rehabilitation, the health condition of elderly patients is unstable and prone to aggravation. Patients in geriatric rehabilitation are characterized by typical geriatric-specific multi-morbidity as well as a high likelihood of development of disease-related complication or medication-related side effects.

### 2.6. Cardiac Rehabilitation

Cardiac rehabilitation aims to facilitate recovery, an increase in physical and mental performance, and to prevent progression of cardiovascular disease.

Cardiac rehabilitation comprises diagnostic and therapeutic approaches and includes components of health education, advice on cardiovascular risk reduction, physical activity, and stress management. Evidence-based preventive measures include pharmacologic and non-pharmacologic intervention. In detail, core components include health behavior change and education, lifestyle risk factor management, psycho-social health, medical risk factor management, cardio-protective therapies, long-term management, and audit and evaluation. Cardiac rehabilitation focuses on patients who are weakened post-intervention (e.g., after heart valve replacement) but present with a Barthel index of at least 70, and thus with a high degree of independence. There are various training methods, such as ergometer and treadmill training, Thera Band ™ (Thera Band ™, Akron, OH, USA) use, strength and muscle building, and swimming.

Often cardiac rehabilitation is divided into three phases with phase I initiated during the index hospital stay focusing on early progressive mobilization of the patient to enabling self-care by discharge, and counseling about the illness, the treatment, risk factors management, and follow-up planning. The next phase is a 3–4-week in-patient rehabilitation program or a supervised ambulatory outpatient program of 3 to 6 months’ duration, which both consist of monitored exercise and further risk factor reduction. Phase III is a lifetime maintenance phase in which physical fitness and additional risk-factor reduction are emphasized.

### 2.7. Statistical Analyses

Data are provided as patient numbers and percentages, normally distributed data are presented as means and SD, and non-normally distributed data as medians with 25–75th percentiles. Data distribution was evaluated using histograms. For comparison of linear variables, one way analysis of variance (ANOVA) or the Mann-Whitney U-test were used. Fisher’s exact test or the chi-square test was used for categorical values as appropriate. A two-sided *p*-value < 0.05 was considered statistically significant. Unadjusted odds ratios (OR) with 95% confidence intervals (95% CI) are provided for mortality and rehospitalization at 6 months. Kaplan-Meier analysis was performed to assess survival during the 6 months after hospital discharge, with a Breslow test used to compare the rehabilitation and no rehabilitation groups. Multivariable analysis of the variables independently associated with mortality at 6 months was performed considering the following variables recorded prior to baseline: age, male sex, body mass index, glomerular filtration rate <60 mL/min, diabetes mellitus, left ventricular ejection fraction, logistic EuroScore I, the use of balloon expandable valves, and the attendance at rehabilitation. The covariates for the regression model were based on statistically significant differences at baseline (threshold *p* ≤ 0.1) as well as on clinical judgement. Statistical analysis was performed using SPSS version 23.0 (IBM Corporation, Amonk, NY, USA).

## 3. Results

### 3.1. Patient Characteristics

**Figure 1:**
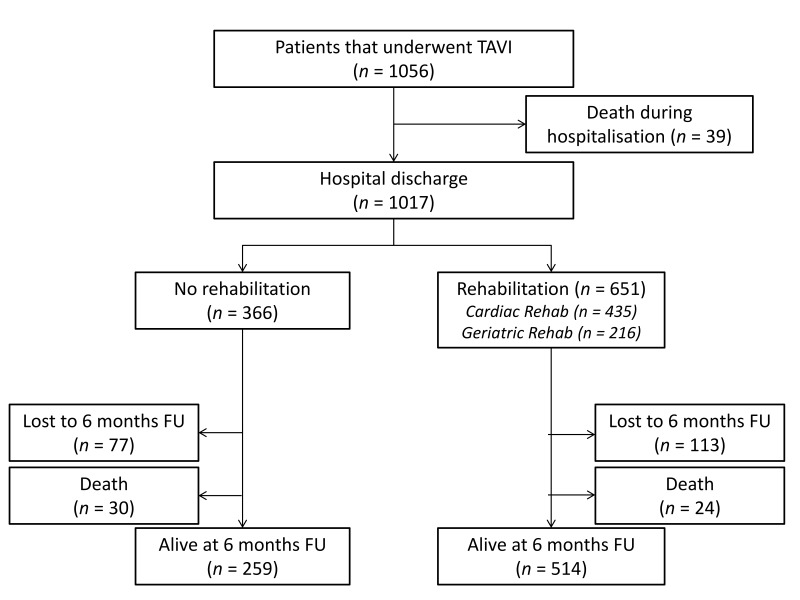
Patient flow through the study. TAVI, transcatheter aortic valve implantation; FU, follow-up.

A total of 1056 patients underwent elective TAVI during the study period. Of these, 1017 survived until hospital discharge and were included in the subsequent analysis (Figure 1). Although rehabilitation was offered to all patients, 366 (36.0%) declined to undergo the program. Of those that chose rehabilitation, 435 (41.8% of the total) underwent a cardiac program and 216 (21.2%) underwent a geriatric program. The majority of patients refusing rehabilitation emphasized the advantages of the home environment and their special life situation.

The baseline characteristics of the patients with and without rehabilitation were similar (Table 1). In accordance with the indications for TAVI, patients were of an advanced age and presented with multiple comorbidities, including coronary artery disease (CAD; 66.1% and 66.0%, respectively; *p* = 0.965) and chronic kidney disease (53.9% and 49.7%, respectively; *p* = 0.199). Diabetes mellitus was less common in patients who underwent rehabilitation than for those who did not (35.5% vs. 46.2%; *p* = 0.001), whereas NT-proBNP levels were lower (2095 vs. 2466 pg/mL; *p* = 0.030).

### 3.2. Procedural and In-Hospital Outcomes

Few patients required conversion to open surgery during the TAVI procedure (0.6% and 1.1% of those with and without rehabilitation, respectively; *p* = 0.467) (Table 2). Similar proportions of the two groups suffered a stroke during the hospital stay (2.0% and 3.3%; *p* = 0.198), whereas pacemaker implantation was more frequently required in those patients who underwent rehabilitation (8.7% vs. 5.8%; *p* = 0.095). The median length of hospital stay after TAVI was longer for the group of patients that went on to undergo rehabilitation (7.0 days; interquartile range (IQR): 6.0, 9.0) than those that declined it (6.0 days; IQR: 5.0, 8.0; *p* < 0.001).

Patient evaluation prior to hospital discharge identified lower NT-proBNP levels than those recorded prior to TAVI, with little variation between the rehabilitation and no rehabilitation groups (1,777 and 1,838 pg/mL, respectively; *p* = 0.334) (Table 2). The mean LVEF was higher in patients that went on to receive rehabilitation (54.2%) than in those who did not (52.5%; *p* = 0.031), whereas the peak valve gradient and levels of aortic insufficiency did not vary significantly.

### 3.3. Outcomes at Six Months

**Figure 2:**
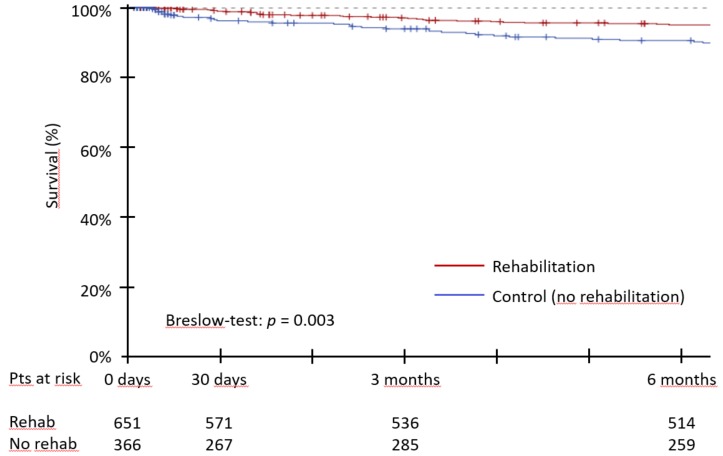
Overall survival during the first six months after TAVI. Kaplan-Meier survival curves for rehabilitation and control (no rehabilitation) groups.

Overall survival during the six months after TAVI was significantly greater for the group of patients that underwent rehabilitation (95.0 vs. 89.8%; *p* = 0.003) (Figure 2).

**Figure 3:**
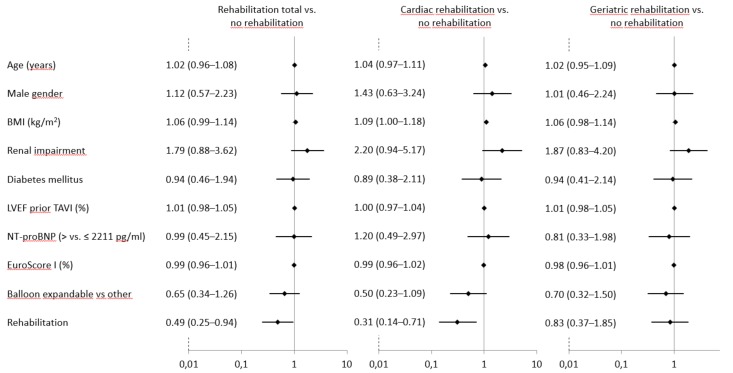
Variables associated with mortality at six months (multivariable adjusted). Legend: BMI, body mass index; renal impairment defined as glomerular filtration rate <60 mL/min; LVEF, left ventricular ejection fraction; TAVI, transcatheter aortic valve implantation; NT-pro BNP values were measured before TAVI.

Upon multivariable analysis (Figure 3), rehabilitation remained associated with a reduced mortality at six months (OR 0.49; 95% CI 0.25–0.94; *p* = 0.032). All other variables were not independent risk factors for the endpoint assessed (age: *p* = 0.57; male sex: *p* = 0.74; BMI: *p* = 0.09; renal impairment defined as GFR < 60 mL/min: *p* = 0.11; LVEF prior TAVI: *p* = 0.36; NT-pro BNP before TAVI: 0.98; EuroScore: *p* = 0.29 and type of valve: *p* = 0.20) (Figure 3).

Assessment of cardiac function at six months identified little variation depending on whether or not patients had undergone rehabilitation (Table 3). Few patients displayed aortic insufficiency and only small proportions of each group were classified as being at NYHA class IV. NT-proBNP levels further decreased in patients with and without rehabilitation and did not differ between both groups at the six-month follow-up time-point. Rehospitalization did not differ between the groups. Patients receiving rehabilitation had lower all-cause mortality at six months, primarily a result of a decreased likelihood on non-cardiovascular mortality (Table 3).

### 3.4. Cardiac vs. Geriatric Rehabilitation

The finding of a reduced mortality being associated with rehabilitation prompted the question of whether a cardiac and geriatric rehabilitation program would potentially differ with respect to outcomes based on their distinct profile.

**Figure 4:**
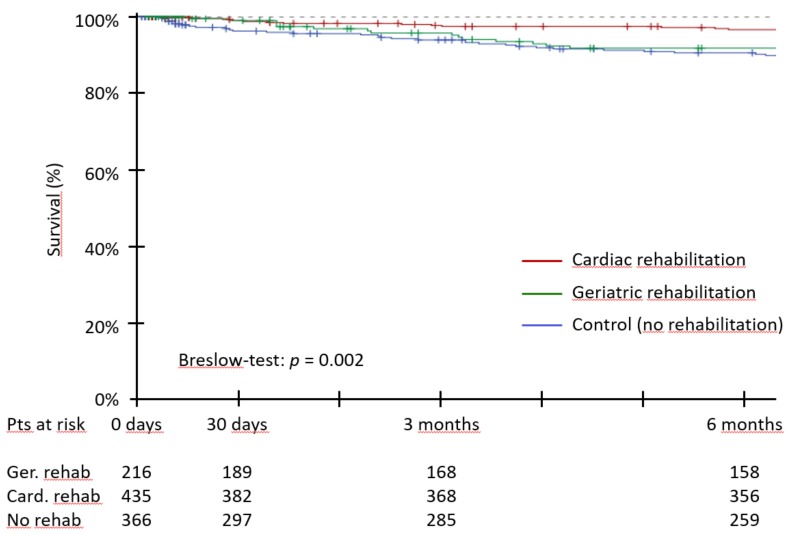
Survival during the first six months after TAVI for patients with cardiac vs. geriatric vs. no rehabilitation. Legend: Kaplan-Meier survival curves for geriatric rehabilitation, cardiac rehabilitation and control (no rehabilitation) groups.

Comparing the survival rate for patients with cardiac, geriatric, or no rehabilitation showed the highest survival rate during the six months after TAVI for the group of patients who underwent cardiac rehabilitation (Figure 4).

Table S1 illustrates that patients undergoing cardiac rehabilitation tended to be younger (80.2 vs. 81.7 years; *p* = 0.003) with more patients that underwent prior CABG (17.8 vs. 9.3%; *p* = 0.004) but with a lower NYHA class (82.0 vs. 88.7%; *p* = 0.049) and logistic EuroScore I (16.0 vs. 19.0%; *p* = 0.005). Cardiac rehabilitation patients had a shorter median hospitalization after TAVI (8 vs. 10 days; *p* < 0.001) and lower levels of NT-proBNP (1063 vs. 2562; *p* < 0.001).

Cardiac rehabilitation was associated with reduced mortality (OR 0.31; 95% CI 0.14–0.71; *p* = 0.006), whereas GFR <60 mL/min was associated with an increased risk (OR 2.20; 95% CI 0.94–5.17; *p* = 0.071) (Figure 3). Geriatric rehabilitation was not independently associated with a change in mortality (OR 0.83; 95% CI 0.37–1.85; *p* = 0.65). There was a non-significant effect of the GFR (OR 1.87; 95% CI 0.83–4.2; *p* = 0.13) and BMI (OR 1.06; 95% CI 0.98–1.14; *p* = 0.12).

## 4. Discussion

In this population of 1017 consecutive patients receiving TAVI, we found six-month survival to be higher in patients that underwent a rehabilitation program after their procedure compared to those that voluntarily declined participation. A cardiac rehabilitation program enforcing physical exercise, in addition to psychosocial training after TAVI, was associated with a higher survival at six months than a geriatric rehabilitation program. The reduced lower all-cause mortality at six months in patients receiving rehabilitation was mainly an effect of a decrease in non-cardiovascular mortality.

The benefits of rehabilitation for patients with CAD, including those undergoing percutaneous coronary intervention (PCI) or coronary artery bypass grafting (CABG), are well-established. Not only have improvements in exercise capacity been demonstrated [10,11] but decreases in mortality have been recorded [12,13]. However, research regarding the benefit of rehabilitation programs in patients with aortic stenosis is scarce. A number of smaller studies have reported improvement in functional and emotional capacity and quality of life for TAVI patients after subsequent rehabilitation programs [4,7,14,15]. However, data about how such changes translate into long-term health outcomes, such as survival, are lacking.

In the present study, superior mortality over the six months after TAVI was observed for the patients that underwent rehabilitation compared to those that declined it. This advantage appeared to be predominantly a result of a decreased likelihood of non-cardiovascular mortality, with the observed reduction in cardiovascular death being less significant. This suggests that improvements in cardiovascular health were not the main driving force behind the lower mortality for the patients that received rehabilitation. This is in agreement with the data regarding cardiac function at six months after TAVI, which showed no significant differences between the two groups. In broad populations of patients with cardiac disease, patients who choose to not participate in cardiac rehabilitation are more likely to be depressed, of low socio-economic status, and physically inactive, predicting poorer clinical outcomes compared to patients receiving rehabilitation [16,17].

Diabetes is an important risk factor for one-year all-cause mortality in patients scheduled for TAVI [18]. In our study, more patients with diabetes and those who were obese refrained from rehabilitation. As patients with diabetes often experience metabolic deterioration after cardiac intervention [19], continuous metabolic monitoring to reduce short- and long-term complications arising from hyperglycemia or hypoglycemia is desirable. It is tempting to speculate that diabetic patients without rehabilitation would have benefited from cardiovascular, metabolic, or musculoskeletal aspects if they had participated in a multicomponent rehabilitation program.

A recent meta-analysis summarizing data from 292 TAVI patients undergoing cardiac rehabilitation program demonstrated improvements in six 6-minute walk distance (6 MWD) and exercise capacity for TAVI patients after rehabilitation [1]. Most of the studies compared post-interventional outcome in patients undergoing TAVI with patients after surgical aortic valve replacement. All studies failed to include a control group consisting of patients that did not undergo such a rehabilitation program [4,7,14,15]. It was therefore impossible to distinguish between the direct effects of rehabilitation and the natural process of recovery with increased physical activity after alleviation of the symptoms of aortic stenosis. Although the changes were found to be statistically significant, the final levels still corresponded to quite poor performance relative to healthy subjects of a similar age [20,21]. Cardiac more than geriatric rehabilitation programs contain multiple components in addition to physical training, including lifestyle counselling and psychological support, providing a variety of benefits for the patient. Frailty, malnutrition, and multimorbidity are common among TAVI patients and are associated with an increased risk of mortality after the procedure [4,5,22,23]. It is therefore possible that the rehabilitation programs utilized in the present study were particularly beneficial for reducing the risk of death due to non-cardiovascular causes, such as infections and accidental injuries. This highlights the importance of evaluating rehabilitation programs for TAVI patients specifically, rather than extrapolating data from studies involving patients with other cardiovascular diseases. Also, the observed longer stay in hospital for patients with geriatric rehabilitation may be explained, at least in part, by legal regulations, as these patients have to be directly transferred from the hospital to a rehabilitation institution, whereas cardiac rehabilitation allows earlier discharge from the hospital. Optimization of rehabilitation strategies specific to the unique needs of TAVI patients may provide even greater benefits than those demonstrated in the present study.

A further notable finding of this analysis was the high proportion of patients that voluntarily declined rehabilitation. Poor uptake of such programs after cardiac surgery has been previously demonstrated [24,25]. Furthermore, Hansen et al. found that TAVI patients were less likely to participate in rehabilitation than those who had surgical valve replacement [26]. Therefore, strategies for improving the uptake of these widely available programs may need to be developed.

### Limitation

The study was designed as a longitudinal observational cohort study with prospective follow-up focusing on six-month mortality. Thus, the results enabled us to generate hypotheses for future research questions when preparing a study with a randomized controlled design. Insufficient information regarding exercise capacity and six MWD was available, in particular, for the patients that did not attend rehabilitation. This prevented comparisons with previous studies that evaluated these outcomes. Also, evaluation of variables, such as frailty, nutritional status, depression, or musculoskeletal status, may have provided further insight into the benefits of the different components of the rehabilitation programs. Patients declining rehabilitation may have been behaviorally and socio-economically different from those who chose to perform rehabilitation, which should be focus in future prospective studies. Health-oriented behavior should be assessed in a controlled design as it was not the primary question of this study.

## 5. Conclusions

Patients that underwent rehabilitation after TAVI had superior overall survival compared to those that voluntarily declined to participate in a program. Tailoring of the components of the rehabilitation to the needs of this unique population, as well as increasing patient participation, could further foster the benefits of TAVI. Future studies should focus on health-oriented behavior as well as identifying risk factors for declining participation in rehabilitation programs.

## Figures and Tables

**Table 1:** Patient characteristics at baseline.

	Rehabilitation (*n* = 651) Mean ± SD or Median (IQR) or proportion of patients (%)	No Rehabilitation (*n* = 366) Mean ± SD or Median (IQR) or proportion of patients (%)	*p*-Value
Age (years)	80.7 ± 6.0	80.1 ± 6.1	0.107
Male sex	290/651 (44.5)	177/366 (48.4)	0.241
Body mass index (kg/m^2^)	27.4 ± 5.3	28.3 ± 6.2	0.017
Diabetes	228/642 (35.5)	166/359 (46.2)	0.001
Chronic kidney disease *	300/651 (53.9)	182/366 (49.7)	0.199
Prior pacemaker	133/644 (20.7)	73/363 (20.1)	0.838
CAD	426/644 (66.1)	235/366 (66.0)	0.965
Prior CABG	96/644 (14.9)	44/356 (12.4)	0.266
Prior PCI	248/644 (38.5)	134/356 (37.6)	0.787
Mitral valve insufficiency (>II°)	22/633 (3.5)	20/356 (5.6)	0.109
Prior valve replacement	24/649 (3.7)	16/364 (4.4)	0.584
LVEF (%)	53.2 ± 12.9	51.7 ± 12.6	0.073
NYHA			0.498
class III	469/620 (75.6)	256/347 (73.8)	
class IV	53/620 (8.5)	35/347 (10.1)	
NT-proBNP (pg/mL)	2095 (871; 5573)	2466 (1117; 6987)	0.030
Logistic EuroScore I (%)	17.0 ± 11.8	17.9 ± 12.6	0.251

Legend: CAD, coronary artery disease; CABG, coronary artery bypass grafting; PCI, percutaneous coronary intervention; LVEF, left ventricular ejection fraction; NYHA, New York Heart Association; NT-proBNP, N-terminal pro brain natriuretic peptide; SD, standard deviation; IQR, interquartile range. * Defined as chronic kidney disease stage 2 or more with glomerular filtration rate (GFR) < 60mL/min.

**Table 2:** Procedural and in-hospital outcomes.

	Rehabilitation (*n* = 651) Mean ± SD or Median (IQR) or proportion of patients (%)	No Rehabilitation (*n* = 366) Mean ± SD or Median (IQR) or proportion of patients (%)	*p*-Value
Conversion to surgery	4/650 (0.6)	4/362 (1.1)	0.467
Stroke	13/649 (2.0)	12/362 (3.3)	0.198
PPI	56/646 (8.7)	21/364 (5.8)	0.095
Valve-in-valve	24/651 (3.7)	15/366 (4.1)	0.743
Hospitalization post-TAVI (days)	7.0 (6.0; 9.0)	6.0 (5.0; 8.0)	<0.001
NT-proBNP (pg/mL)	1777 (775; 3,722)	1838 (879; 4,232)	0.334
Cardiac function at discharge ^†^			
LVEF (%)	54.2 ± 11.1	52.5 ± 11.7	0.031
Peak valve gradient (mmHg)	11.8 ± 5.9	11.7 ± 5.5	0.865
Aortic insufficiency			0.230
mild	25/581 (4.3)	9/321 (2.8)	
moderate/severe	1/581 (0.2)	2/321 (0.6)	

Legend: PPI, permanent pacemaker implantation; LVEF, left ventricular ejection fraction; NT-proBNP, N-terminal pro brain natriuretic peptide. SD, standard deviation; IQR, interquartile range. ^†^ Discharge from department where the TAVI was performed (discharge to home or rehabilitation unit).

**Table 3:** Outcomes and cardiac function at six months.

	Rehabilitation Mean ± SD or Median (IQR) or proportion of patients (%)	No Rehabilitation Mean ± SD or Median (IQR) or proportion of patients (%)	*p*-Value
All-cause mortality	24/538 (4.5)	30/289 (10.4)	0.001
CV death	9/538 (1.7)	8/289 (2.8)	0.290
Non-CV death	7/538 (1.3)	14/289 (4.8)	0.002
Unknown cause of death	8/538 (1.5)	8/289 (2.8)	0.202
Rehospitalization	93/538 (17.3)	61/289 (21.1)	0.178
Cardiac function			
LVEF (%)	55.0 ± 10.3	54.6 ± 10.6	0.636
Peak valve gradient (mmHg)	11.2 ± 5.7	11.3 ± 5.4	0.982
Aortic insufficiency			0.287
mild	17/414 (4.1)	6/222 (2.7)	
moderate/severe	1/414 (0.2)	2/222 (0.9)	
NYHA			0.105
Class III	158/392 (40.3)	76/206 (36.9)	
Class IV	4/392 (1.0)	7/206 (3.4)	
NT-proBNP (pg/mL)	849 (364, 1952)	758 (381, 1951)	0.778

Legend: LVEF, left ventricular ejection fraction; NYHA, New York Heart Association; NT-proBNP, N-terminal pro brain natriuretic peptide; CV, cardiovascular; SD, Standard Deviation; IQR, Interquartile ranges.

## Supplementary Materials

The following are available online at http://www.mdpi.com/2077-0383/7/10/326/s1, Table S1: Patient characteristics at baseline.
