# Supplementary material for: Impact of Rehabilitation on Outcomes after TAVI: A Preliminary Study
Source: J Clin Med. 2018 Oct 5;7(10):326. doi: 10.3390/jcm7100326 (PMC6210128; doi:10.3390/jcm7100326)
Supplement: Supplementary file 1 [file jcm-07-00326-s001.pdf]

Supplement

Table S1. Patient characteristics at baseline

	Cardiac rehabilitation (n = 435) Mean ± SD or median (IQR) or n/N (%)	Geriatric rehabilitation (n = 216) Mean ± SD or median (IQR) or n/N (%)	P-value
Age (years)	80.2 ± 6.2	81.7 ± 5.3	0.003
Male gender	204/435 (46.9)	86/216 (39.8)	0.087
Body mass index (kg/m^2)	27.2 ± 4.9	27.9 ± 6.1	0.181
Diabetes	143/431 (33.2)	85/211 (40.3)	0.077
Chronic kidney disease*	193/435 (33.3)	107/216 (49.5)	0.213
Prior pacemaker	90/433 (20.8)	43/211 (20.4)	0.905
CAD	291/428 (68.0)	135/216 (62.5)	0.164
Prior CABG	76/428 (17.8)	20/216 (9.3)	0.004
Prior PCI	168/428 (39.3)	80/216 (37.0)	0.585
Mitral valve insufficiency (>II°)	12/422 (2.8)	10/211 (4.7)	0.220
Prior valve replacement	20/435 (4.6)	4/214 (1.9)	0.083
LVEF (%)	53.5 ± 12.7	52.7 ± 13.3	0.454
NYHA			0.049
class III	309/415 (74.5)	160/205 (78.0)	
class IV	31/415 (7.5)	22/205 (10.7)	
NT- proBNP (pg/ml)†	1904 (809; 4743)	2642 (1137; 7320)	0.002
Log. EuroSCORE I (%)	16.0 ± 11.0	19.0 ± 13.1	0.005
Conversion to surgery	2/435 (0.5)	2/215 (0.9)	0.603
Stroke	9/434 (2.1)	4/215 (1.9)	1.000
PPI	32/433 (7.4)	24/213 (11.3)	0.100

Valve-in-valve	18/24 (4.1)	6/216 (2.8)	0.386
Hospitalization post-TAVI (days)	8.0 (6.0; 10.9)	10.0 (8.0; 16.0)	<0.001
NT-proBNP (pg/ml)	1603 (706; 3175)	2562 (1049; 5023)	<0.001
Cardiac function at discharge†			
LVEF (%)	54.2 ± 10.8	53.9 ± 11.8	0.681
Peak valve gradient (mmHg)	11.9 ± 5.9	11.7 ± 6.0	0.752
Aortic insufficiency			0.887
mild	16/386 (4.1)	9/195 (4.6)	
moderate/severe	1/386 (0.3)	0/195 (0)	

Legend: CAD, coronary artery disease; CABG, coronary artery bypass grafting; PCI, percutaneous coronary intervention; LVEF, left ventricular ejection fraction; NYHA, New York Heart Association; NT-proBNP, N-terminal pro brain natriuretic peptide; SD, standard deviation. *Defined as chronic kidney disease stage 2 or more with GFR < 60 ml/min; PPI, permanent pacemaker implantation; LVEF, left ventricular ejection fraction †Discharge from department where the TAVI was performed (discharge to home or rehabilitation unit).
